# Overexpression of TNKS1BP1 in lung cancers and its involvement in homologous recombination pathway of DNA double‐strand breaks

**DOI:** 10.1002/cam4.995

**Published:** 2017-01-06

**Authors:** Wei Tan, Hua Guan, Lian‐Hong Zou, Yu Wang, Xiao‐Dan Liu, Wei‐Qing Rang, Ping‐Kun Zhou, Hua‐Dong Pei, Cai‐Gao Zhong

**Affiliations:** ^1^ XiangYa School of Public HeathCentral South UniversityChangshaHunan Province410078China; ^2^ Beijing Key Laboratory for RadiobiologyDepartment of Radiation Toxicology and OncologyBeijing Institute of Radiation MedicineBeijing100850China; ^3^ Institute for Environmental Medicine and Radiation HygieneThe College of Public HealthUniversity of South ChinaHengyangHunan Province421000China; ^4^ State Key Laboratory of ProteomicsBeijing Proteome Research CenterBeijing Institute of Radiation MedicineBeijing100039China; ^5^ National Center for Protein Sciences (The PHOENIX center, Beijing)Beijing102206China

**Keywords:** Lung adenocarcinoma (LAC), chemotherapy, cell cycle, DNA homologous recombination, TNKS1BP1

## Abstract

TNKS1BP1 is a member of the poly(ADP‐ribose) polymerase (PARP) superfamily. Our previous studies have demonstrated that TNKS1BP1 plays an important role in DNA damage response. But whether and how TNKS1BP1 associates with cancer is still not clear. Here, we found that TNKS1BP1 was upregulated in human lung adenocarcinoma (LAC) tissues, and was associated with poor overall survival (OS) in LAC patients. Dysregulation of TNKS1BP1 affected the sensitivity of A549 cells to several DNA damage agents including cisplatin, bleomycin, and ionizing radiation. Mechanically, overexpression of TNKS1BP1 increased the accumulation of S phase cells, which was accompanied by a decrease in M phase cells. More importantly, we found TNKS1BP1 regulated genome stability, mainly through affecting the homologous recombination pathway of DNA double‐strand breaks by inhibiting the RAD51 foci formation. Overall, our study indicates that, in LAC, aberrant expressions of TNKS1BP1 are common events, and overexpression of TNKS1BP1 might affect outcomes of cancer patients to chemotherapy and radiotherapy.

## Introduction

TNKS1BP1, a tankyrase 1 binding protein 1 of 182 kDa, also named TAB 182, has been identified in a yeast two‐hybrid experiment as a binding protein of tankyrase 1, which was found to poly(ADP‐ribosyl)ate TNKS1BP1 in vitro. *TNKS1BP1* encodes a protein of 1729 amino acids with a large internal acidic region (200‐1572aa) containing a RXXPDG motif 1 that is necessary and sufficient for tankyrase binding. TNKS1BP1 localizes to cytoplasm in which it co‐stains with the cortical actin network and nucleus in a heterochromatic staining pattern. 2. Previous studies have demonstrated that TNKS1BP1 functions in both telomere maintenance and DNA damage response. TNKS1BP1 can compete with TRF1 to link the multiple binding site of tankyrase1 which potentially interferes with the transport of tankyrase 1 from cytoplasm to nucleus and the location in telomeres, affecting the stability of telomeres 2, 3. And *TNKS1BP1* expression could be upregulated by *γ*‐ray irradiation, and depletion of TNKS1BP1 was found to sensitize HeLa cells to the ionizing radiation (IR)^4^.However, whether TNKS1BP1 functions in tumorigenesis and cancer cellular response to radiation and chemotherapy is still unclear.

The aim of this study was to investigate the expression status of TNKS1BP1 in lung adenocarcinoma tissues and its possibility as a biomarker of cancer cells. The potential influence of TNKS1BP1 on cancer cellular response to chemotherapy was also studied. To address these issues, the expression patterns of TNKS1BP1 in human clinical lung adenocarcinoma tissues were examined, and the analysis of patient survival data was used to evaluate their prognostic values. And we also try to figure out the effects of TNK1SBP1 cancer cellular response to chemotherapy and tumorigenesis.

## Materials and Methods

### Cell culture, plasmids, and reagents

A549 cell line was obtained from ATCC and maintained in normal medium of RPMI 1640(Hyclone) containing 10% fetal bovine serum (FBS, Gibco), penicillin (100 U/mL), and streptomycin (100 mg/mL) at 37°C in a 5% CO2 incubator. The pSicoR‐puro‐shTNKS1BP1 plasmid containing the sequence of shRNA was:5′‐TGGAGAGTTCCTTAAATCAAGGTTCAAGAGACCTTGATTTAAG GAACTCTCCTTTTTTC‐3′(sense). PLPC‐Myc‐TNK1BP1 expression vector was kindly supplied by Dr. Susan Smith (Kimmel Center for Biology and Medicine of the Skirball Institute, New York University School of Medicine, USA) 2. Primary antibodies used in this study were as follows: anti‐TNKS1BP1, anti‐CtIP and BRCA1 (Santa Cruz), anti‐TNKS1BP1, anti‐Ku70 and RAD51 (Gene Tex), Anti‐pH3 and anti‐*γ*H2AX (Millipore), anti‐53BP1 (Cell Signalling Technology). homologous recombination (HR) and Nonhomologous end joining (NHEJ) reporter plasmids were gifted by Dr Zhenkun Lou (Division of Oncology Research, Mayo Clinic Rochester, USA).

### Immunohistochemistry

The lung adenocarcinoma (LAC) tissues’ microarrays were purchased from the National Engineering Center for BioChips in Shanghai, China 5. The expression of TNKS1BP1 was evaluated by immunohistochemical staining with a TNKS1BP1‐specific antibody (GTX108091). Each sample was scored according to the percentage of positive‐stained cells and staining intensity. The final immunoreactivity score was calculated by multiplying the product of staining intensity by the percentage of stained cells 6.

### Cell viability assay

Cisplatin and bleomycin were purchased from sigma and Nippon Kayaku Co Ltd, respectively. A549 cells were planted on 96‐well plates (2000 cells per well) and allowed to grow for 24 h. The cells were then exposed to drugs tested. After 48‐h incubation, the absorbance values (OD) were measured at 450 nm by UV‐Visible Spectrophotometer. The rate of cell viability was calculated as follow: [OD of experiment well – OD of blank well]/[OD of control well – OD of blank well] × 100%. Data were presented as mean ± *SEM* of three independent experiments at least.

### Apoptosis assay

Cells were identified by dual staining with PI and FITC‐labeled Annexin V following the manufacturer's guidelines (BD Biosciences) and were analyzed by the flow cytometry (BD Biosciences).

### Cell cycle analysis

The cells were harvested and fixed by 70% ethanol, washed by PBS and permeabilized by 0.2% TritonX‐100 for 15 min on ice. The samples were washed with 1 × PBS again and incubated with the anti‐phospho HistoneH3^S10^ 488‐conjugated antibody (1:100) for 1 h at room temperature 7. Pellet cells were washed twice with PBS. DAPI staining at 0.2–0.5 *μ*g per ml was performed to visualize nuclear DNA. All the samples were measured on BD LSRFortessa™ flow cytometer.

### Immunofluorescence Staining

Immunofluorescence staining was performed as previously described 8. Briefly, cells were fixed in 3% paraformaldehyde and permeabilized in 0.5% Triton X‐100 solution. Then they were blocked and incubated with indicated antibody. Nuclear DNA was stained with DAPI. The Nikon ECLIPSE Ti fluorescence microscope was used to visualize the samples.

### Colony formation Assay

The cells were seeded on 60‐mm plates and grew for 12 h, then irradiated at different does of 0, 2, 4, 8 Gy of X‐ray. After culturing for 10 days, the cell colonies were fixed with 70% ethanol and stained with Giemsa solution. The colonies with more than 50 cells were calculated.

### DNA double‐strand breaks repair assay

To measure the double‐strand break (DSB) repair, A549 cells were planted on 35‐mm plates and transfected the next day with 7 *μ*L of 20 *μ*mol/L siRNA mixed with 7.5 *μ*L Lipofectamine® RNAiMAX reagents in Optimem. After 8 h, the transfected complexes were changed into normal medium. The third day, cells were transfected with equal siRNA repeatedly. On the fourth day, the cells were transfected with HR or NHEJ report vectors 9. After 36 h (NHEJ) or 48 h (HR) of transfection, cells were harvested and analyzed by FACS as previously described 8. The repair efficiencies of HR and NHEJ were, respectively, calculated as following: the efficiency (%) = [Q2/(Q1 + Q2)] ×100%. After normalization, the efficiency of experiment group was presented as the percentage of control.

### Statistical analysis

SPSS version 13.0 was used for all statistical analysis. Values were shown as mean ± *SEM*. The Wilcoxon test was used to analyze the protein expression in lung cancer tumors and paired adjacent normal tissues. The overall survival curves was used in Kaplan–Meier method and compared between groups used the log‐rank test. The one‐way ANOVA was used to analyze the inhibition rate between different treatment groups. Bonferroni was used for multiple comparisons. Student's t test was used for two independent sample comparisons. All statistical tests were two‐sided and significance was set at *P *<* *0.05.

## Results

### Overexpression of TNKS1BP1 in lung adenocarcinoma and its correlation with the patient survival

**Figure 1:**
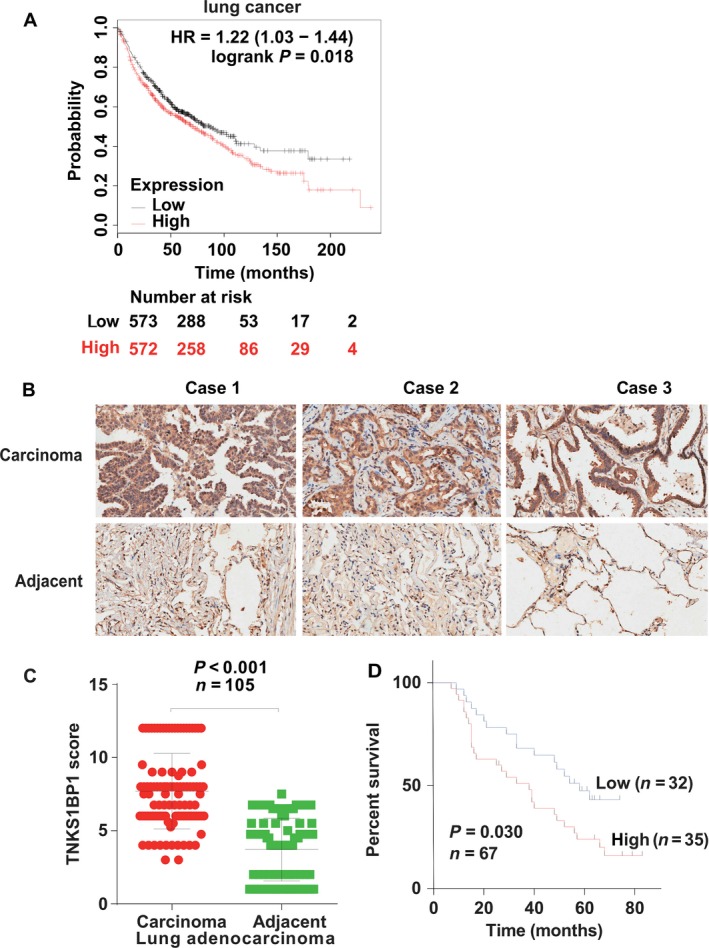
TNKS1BP1 is overexpressed in lung adenocarcinoma (LAC) and correlated with the patient survival. (A) Higher *TNKS1BP1* expression was correlated with poor outcomes in lung cancer patients. (B) Representative image for immunohistochemical staining of TNKS1BP1 expression in the tumors (lung cancer) and adjacent normal tissues from three cases. (C) Box plot of TNKS1BP1 expression in lung cancer from 105 cases. Expression scores were compared between tumors and matched adjacent normal tissue using the Wilcoxon test (two‐sided, *n* = 105, *P *<* *0.001). (D) Kaplan–Meier estimates of the cumulative survival rate (two‐sided, *n* = 67, *P *=* *0.030).

TNKS1BP1 was expressed abundantly in multiple tissues, such as lung, ovary, and testis 2. To assess whether TNKS1BP1 plays an important role in cancer development, we analyzed *TNKS1BP1* expression pattern in the lung cancer and ovarian cancer at genomic level based on the public database (http://kmplot.com/analysis/). We found that higher *TNKS1BP1* expression was correlated with poor outcomes in lung cancer patients (Fig. 1A) rather than in ovarian cancer (Fig. S1). To further confirm this result, we performed the immunohistochemical staining (IHC) analysis to score the expression of TNKS1BP1 in 105 lung adenocarcinoma tissue samples. As shown in Figure 1B and 1C, TNKS1BP1 proteins were observed distributed in both cytoplasm and nucleus of LAC cells as well as adjacent noncancerous epithelial cells. The majority of the tumor tissues showed high expression levels of TNKS1BP1. In marked contrast, the expressions of TNKS1BP1 were significantly decreased in the noncancerous tissues. In addition, the intensity staining score in the tumor tissue is much higher than that in the normal tissue (Fig. 1C). The prognostic value of TNKS1BP1 protein expression in LAC patients was then determined. The Kaplan–Meier analysis showed that the overall survival (OS) rates of patients with high TNKS1BP1 were significantly lower than that of patients with low TNKS1BP1. The 5‐year OS rates for patients with high and low levels of TNKS1BP1 were 22.86% and 37.50%, respectively. These results imply that TNKS1BP1 could be a novel indicator for the diagnosis and treatment of LAC patients in the future.

### Effect of TNKS1BP1 on cellular sensitivity to DNA damage agents

**Figure 2:**
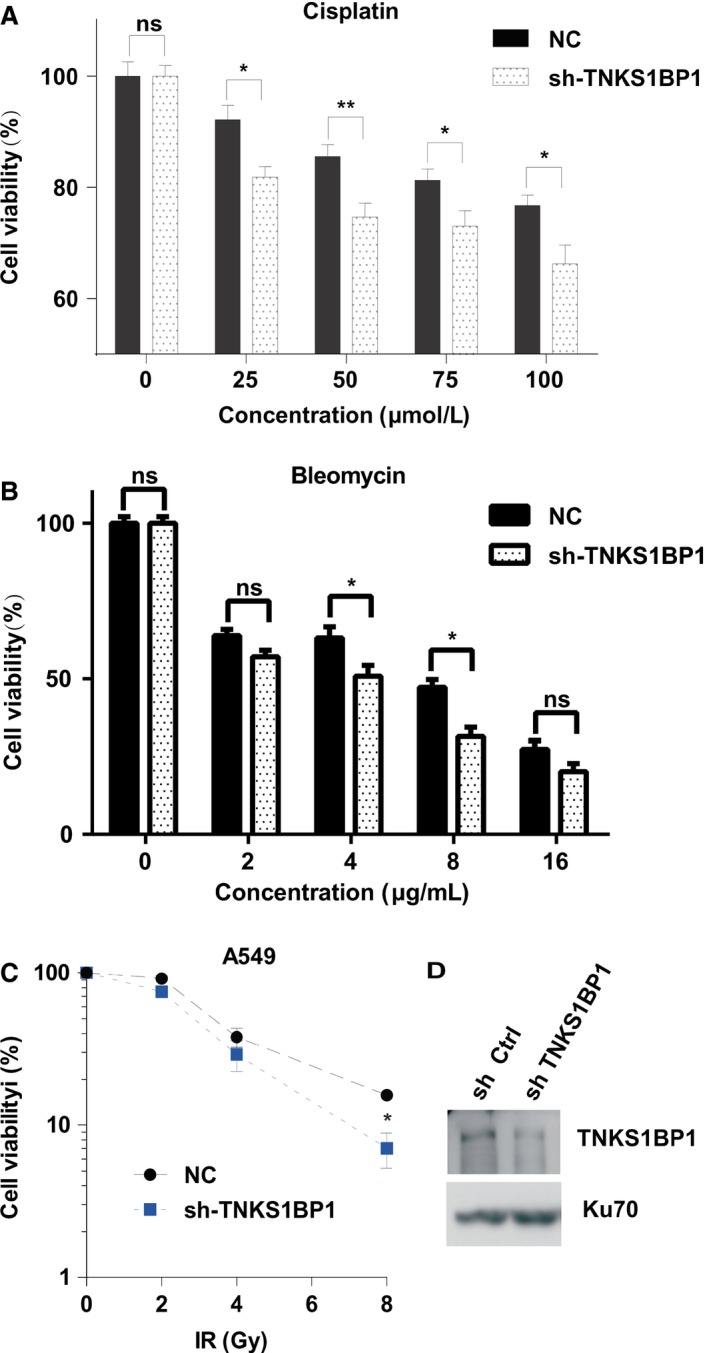
Effects of TNKS1BP1 on cellular response to DNA damage agents. (A–B) CCK8 assay of A549 cells with TNKS1BP1 deficiency compared with control after exposing cisplatin (A) and bleomycin (B). The error bars represent standard error of mean (*SEM*) from more than three independent experiments. The one‐way ANOVA and Bonferroni were used to compare the quantitative data among different groups and multiple comparisons, *P <* *0.05, **P <* *0.01. (C) Knockdown of TNKS1BP1 by shRNA (sh‐TNKS1BP1) sensitized cells to X‐ray irradiation as compared to the control cells. Data were analyzed by one‐way ANOVA (*n* = 3, *P <* *0.05). (D) Knockdown efficiency of TNKS1BP1 by shRNA was confirmed by western blotting analysis.

To examine whether TNKS1BP1 affects cellular response to chemotherapeutic drugs, we tested the influence of TNKS1BP1 on the sensitivity of human lung cancer cell line, A549 to two DNA damage reagents cisplatin and bleomycin. Downregulation of TNKS1BP1 with the specific shRNA resulted in decreased resistance of A549 cells to these treatments (Fig. 2A–B). Overall, these results have demonstrated that TNKS1BP1 might play an important role in regulating cellular response to the main anti‐neoplastic agents commonly used in clinical lung cancers treatments.

The effect of TNKS1BP1 on cellular radiosensitivity has been further investigated using the colony formation assay after X‐ray irradiation, a typical physical DNA damage agent. We found that TNKS1BP1 depletion also sensitized cells to the DNA damage induced by ionizing radiation (Fig. 2C–D).

### TNKS1BP1 affects cell cycle distribution

**Figure 3:**
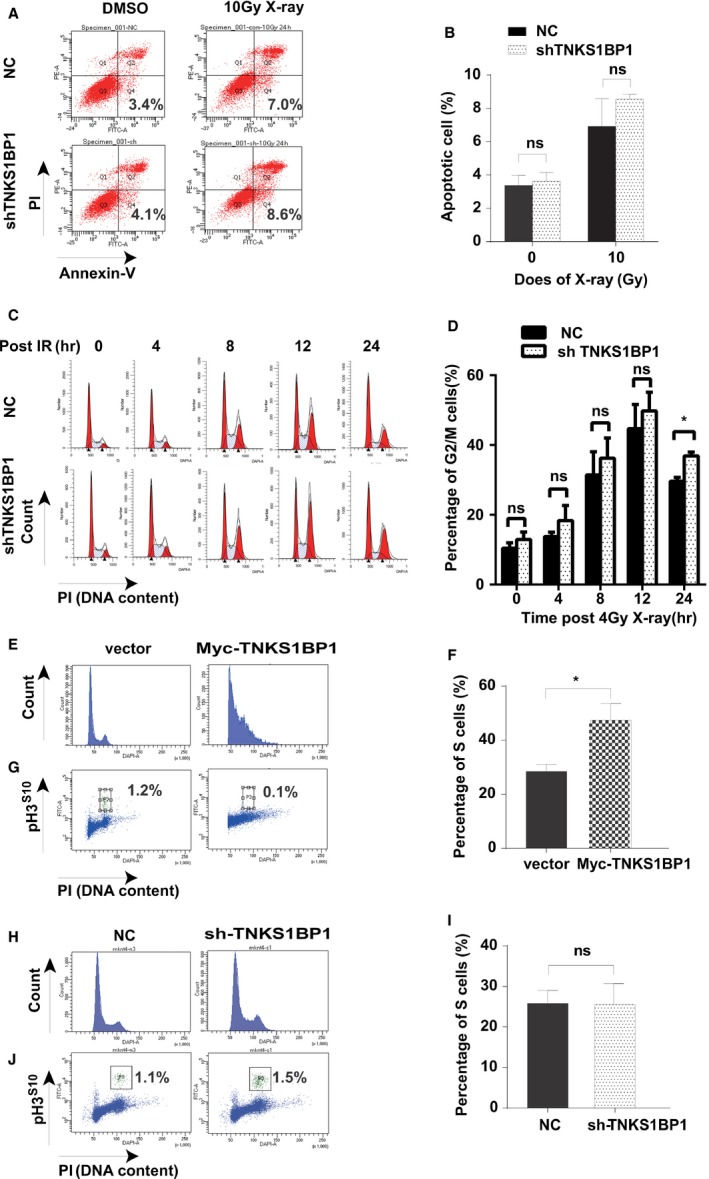
TNKS1BP1 overexpression results in S phase cells accumulation. (A–B) Depletion of TNKS1BP1 does not markedly regulate the apoptosis after 10 Gy of X‐ray irradiation in A549 cells. The Y axis shows the PI staining of genomic DNA and X axis shows the annexie V‐FITC staining cells. Data were presented as mean±*SEM* from two independent experiments. Data were analyzed by Student's *t* test (*n* = 2, two‐sided, *P *>* *0.05). (C–D) Depletion of TNKS1BP1 can extend the G2/M arrest after 4 Gy of X‐ray irradiation. Data were presented as mean ± *SEM* from three independent experiments. Data were analyzed by Student's *t* test (*n* = 3, two‐sided, *P <* *0.05). (E–F) Flow cytometry analysis of cycle alterations caused by overexpressing TNKS1BP1. Data were presented as mean ± *SEM* from more than three independent experiments. Data were analyzed by Student's *t* test (*n* = 6, two‐sided, *P <* *0.05). (G) Measurement of mitotic cells by Flow cytometry with PI and pH3^S10^staining in A549 cells with overexpressed TNKS1BP1. (H–I) Flow cytometry analysis of TNKS1BP1 depleted A549 cells and control cells. Data were presented as mean ± *SEM* from three independent experiments. Data were analyzed by Student's *t* test (*n* = 3, two‐sided, *P *>* *0.05). (J) Measurement of mitotic cells by Flow cytometry with PI and pH3^S10^staining in A549 cells with depletion of TNKS1BP1.

Next, we plan to figure out how does TNKS1BP1 regulate cellular response to these DNA damage agents? First, we checked if TNKS1BP1 is involved in apoptosis pathway. As shown in Figure 3A and B, knockdown of TNKS1BP1 did not increase the occurrence of apoptosis after 10 Gy of X‐ray irradiation. Then the cell‐cycle distributions were further detected. As shown in Figure 3C–D, we found depletion of TNKS1BP1 enhanced the G2/M arrest and made A549 cells to be released lately from the IR‐induced G2‐M arrest. Moreover, overexpression of TNKS1BP1 largely increased the ratio of S phase cells even without irradiation (Fig. 3E–F), which was accompanied by a significant decrease in M phase cells (Fig. 3G). But the ratio of S phase was not significantly changed by the depletion of TNKS1BP1 (Fig. 3H–J). These data indicated that TNKS1BP1 overexpression affects the normal cell division and growth.

### TNKS1BP1 regulates DNA Homologous Recombination

**Figure 4:**
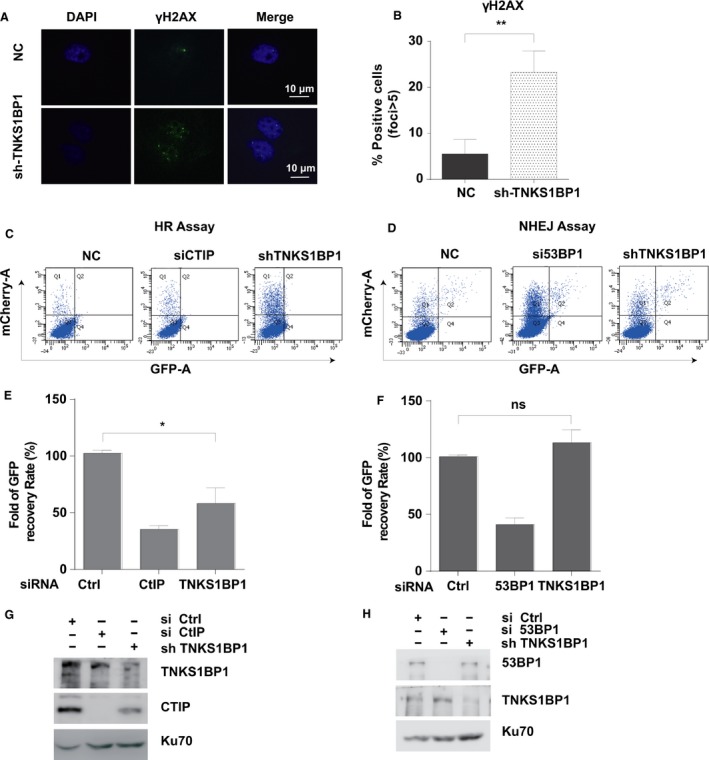
TNKS1BP1 regulates DNA Homologous Recombination. (A) Immunofluorescence staining showed that TNKS1BP1 depletion resulted in *γ*H2AX foci formation (left). (B) Quantification result of *γ*H2AX foci. The data were presented as the mean ± *SD* from two independent experiments. More than 50 cells were counted in each experiment. Data were analyzed by Student's *t* test (*n* = 2, two‐sided, *P <* *0.05). (C–D) FACS detected the effect of TNKS1BP1 depletion on the homologous recombination (HR) and nonhomologous end joining (NHEJ) efficiency. TNKS1BP1 was depleted with targeting shRNA in A549 cells integrated with HR or NHEJ reporter. Cells reconstituted with the indicated reporter were subjected to HR assay (C) and NHEJ assay (D) as described in Materials and Methods. (E–F) Data of HR(C) and NHEJ(D) assays were presented as the mean±*SEM* from two independent experiments. Data were analyzed by Student's *t* test (*n* = 2, two‐sided, *P <* *0.05). (G–H) Knockdown efficiencies of (C) and (D) were, respectively, confirmed by western blotting analysis.

As shown in Figure 2C, depletion of TNKS1BP1 sensitized A549 cells to ionizing radiation. It is well known that DNA DSB is a major type of DNA damage which determines the sensitivity of cells to ionizing radiation. At DSB sites, *γ*H2AX foci are quickly formed and persist if DSBs are not repaired 8, 10. To confirm the role of TNKS1BP1 in genome stability maintenance, we examined *γ*H2AX foci formation in TNKS1BP1‐depleted cells even without DNA damage treatment. As shown in Figure 4A–B, depletion of TNKS1BP1 resulted in elevated levels of spontaneous *γ*H2AX foci formation, suggesting that TNKS1BP1 contributes to maintenance of genome integrity.

**Figure 5:**
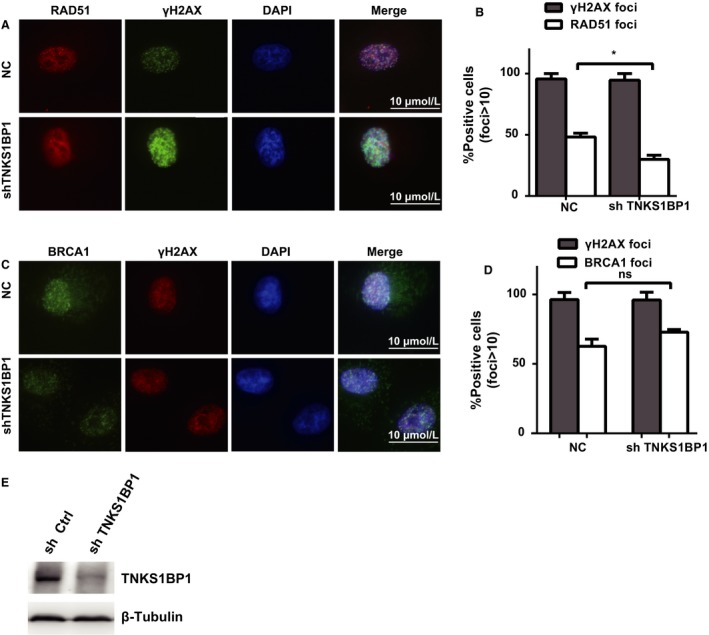
Depletion of TNKS1BP1 inhibits RAD51 foci formation. (A‐B) TNKS1BP1 is required for RAD51 foci formation. TNKS1BP1‐depleted cells were treated with ionizing radiation (10 Gy) and allowed to recover for 2 h before fixing and processing for RAD51 immunofluorescence. The data were presented as the mean ± *SEM* from two independent experiments. More than 50 cells were counted in each experiment. Data were analyzed by Student's *t* test (*n* = 2, two‐sided, *P <* *0.05). (C–D) TNKS1BP1 is not required for BRCA1 foci formation. TNKS1BP1‐depleted cells were treated with ionizing radiation (10 Gy) and allowed to recover for 2 h before fixing and processing for BRCA1 immunofluorescence. The data were presented as the mean ± *SEM* from two independent experiments. More than 50 cells were counted in each experiment. Data were analyzed by Student's *t* test (*n* = 2, two‐sided, *P *>* *0.05). (E) Depletion efficiency of TNKS1BP1 by shRNA was confirmed by western blotting analysis.

NHEJ and HR are two key pathways that repair DSB 11, 12, 13. By the reporter assays for NHEJ and HR 8, 9, the depleting TNKS1BP1 decreased the HR efficiency to the extent similar to that reached by knocking down the key HR factor CTIP 14, 15, 16 (Fig. 4C, E, G). Conversely, we found the repair efficiency affected in TNKS1BP1‐depleted cells was different to that achieved by depleting the key NHEJ factor 53BP1 17, 18, 19 (Fig. 4D, F, H). So, we further checked some markers such as BRCA1 20, 21, 22 and RAD51 23 in HR pathway foci formation in TNKS1BP1‐depleted cells. The results showed depletion of TNKS1BP1 dramatically inhibited RAD51 foci formation (Fig. 5A–B) except for BRCA1 (Fig. 5C–E). We further detected whether TNKS1BP1 affected the cell cycle without DNA damage. First, we found overexpression of TNKS1BP1 increased the ratio of S phase without DNA damage and the BRCA1 foci in early time points after irradiation (Fig. S2A–B). On the other hand, depletion of TNKS1BP1 had no significant effect on the S phase in A549 cells without DNA damage (Fig. 3H–J), indicating that TNKS1BP1 function in HR was not caused by the cell cycle change.

## Discussion

Lung cancer is the most lethal malignant tumor and common cause of death from carcinoma globally and NSCLC is nearly larger than 80% of the lung cancer 24, 25. The chemotherapy of DNA damage agents is considered as an important type of treatment of all stage cancer to improve the patients’ survival 26. But the lung cancer remains incurable since the carcinomas that are sensitive to chemotherapy could rapidly develop acquired drug resistance 27. While the lung cancer as a metastatic cancer exhibits multiple and heterogeneous genomic alterations, individualized treatments are needed to attenuate the resistance 24, 28. So, it is important to find new biomarkers or indicators for lung cancer diagnosis and prognosis judgment of chemotherapy or radiotherapy. Our results indicated that TNKS1BP1 is implicated in the prognosis and outcome of cancer. TNKS1BP1 is upregulated in LAC and may confer lung cancer cells resistance to chemotherapy and radiotherapy.

Mechanically, we found that TNKS1BP1 affected both of cell cycle distribution and genome stability But the detailed mechanism is still not completely clear. Many cell regulators and DNA damage response players, such as cyclinD1 29, 30, PLK1 31, 32 and BRCA1 20, 33, are mutated or dysregulated in cancers. It is plausible that upregulation or mutation of TNKS1BP1 may contribute to not only chemoresistance but also tumorigenesis. We found that TNKS1BP1 expression is high in lung cancers, supporting a possible role of TNKS1BP1 in tumorigenesis. However, the causal role of TNKS1BP1 in tumorigenesis remains to be determined.

In eukaryotic cells, NHEJ and HR are two key pathways that mediate DSB repair 11, 12, 13. NHEJ repairs DSBs by the re‐ligation of broken DNA ends without the participation of homologous DNA. NHEJ is considered error‐prone and mutagenic given that a homologous template is not needed to guide repair. HR is considered an error‐free mechanism for DSB repair and restricted in S‐ and G2‐phase of cell cycle because it needs to employ homologous sequence in the sister chromatid as a template to prime repair synthesis and restore chromosome integrity 34. It is affected throughout the cell cycle by regulating the expression of its key factors: BRCA1, BRCA2, RAD51, CTIP and so on 34, 35, 36, 37, 38. 53BP1 can inhibit the BRCA1 function by impairing the 5′ end resection needed for HR to promote the NHEJ 19. Depletion of TNKS1BP1 increased the *γ*H2AX foci formation without the DNA damage treatment; thus, we confirm the role of TNKS1BP1 in genome stability maintenance. By the DNA repair assay, TNKS1BP1 depletion decreased the HR efficiency, but had no significant effect on NHEJ pathway. Importantly, knockout of TNKS1BP1 had no significant effect on the cell cycle and BRCA1 foci formation, but RAD51 foci were significantly decreased. These data indicated that depletion of TNKS1BP1 regulated HR not through the cell cycle change, but depended on inhibiting the RAD51 foci formation.

Therefore, TNKS1BP1 is a novel regulator of DNA double‐strand breaks in HR pathway by inhibiting the RAD51 foci formation. Of course, the further mechanism of TNKS1BP1 function for it remains to be uncovered in the future.

## Conclusion

TNKS1BP1 is overexpressed in human lung adenocarcinoma (LAC) and correlated with the LAC patients’ survival. TNKS1BP1 presents a potential new biomarker for LAC. TNKS1BP1 regulates the genomic stability and cellular sensitivity to DNA damage agents in A549 cells, and is associated with its function in regulating HR repair by inhibiting the RAD51 foci formation.

## Conflict of Interest

None declared.

## Supporting information


**Figure S1.** TNKS1BP1 expression was correlated with outcomes in ovarian cancer.

**Figure S2.** TNKS1BP1 overexpression increases the BRCA1 foci. (A) BRCA1 foci formation following DNA damage was increased in TNKS1BP1 overexpression cells. The cells were treated with ionizing radiation (10 Gy) and allowed to recover for indicating time points. The data were presented as the mean±*SEM* from two independent experiments. More than 50 cells were counted in each experiment. Data were analyzed by Student's *t* test (*n* = 2, two‐sided, *P <* *0.05, **P <* *0.01). (B) TNKS1BP1 overexpression was confirmed by western blotting analysis in A549 cell.
