# EXPRESSION OF CONCERN: Overexpression of TNKS1BP1 in Lung Cancers and Its Involvement in Homologous Recombination Pathway of DNA Double‐Strand Breaks

**DOI:** 10.1002/cam4.71947

**Published:** 2026-05-15

**EXPRESSION OF CONCERN:** W. Tan, H. Guan, L.‐H. Zou, Y. Wang, X.‐D. Liu, W.‐Q. Rang, P.‐K. Zhou, H.‐D. Pei, and C.‐G. Zhong, “Overexpression of TNKS1BP1 in Lung Cancers and Its Involvement in Homologous Recombination Pathway of DNA Double‐Strand Breaks,” *Cancer Medicine* 6, no. 2 (2017): 483–493, https://doi.org/10.1002/cam4.995.

This Expression of Concern is for the above article, published online on 06 January 2017 in Wiley Online Library (wileyonlinelibrary.com), and has been issued by agreement between the journal Editor‐in‐Chief, Stephen Tait; and John Wiley & Sons Ltd. Concerns were raised by a third party on PubPeer [1] regarding duplication and cropping of the Ku70 bands in Figures 2C and 4H, even though both figures represent different experimental conditions.

An investigation by the publisher confirmed the duplication. The authors did not respond to an inquiry and request for original data by the publisher. Because this duplication may affect interpretation of the results in the article, the Expression of Concern has been agreed to in order to inform and alert readers about the concerns in Figures 2C and 4H. The authors were informed of this Expression of Concern.

**References**

[1] Aneurus Inconstans, Comment on “Overexpression of TNKS1BP1 in lung cancers and its involvement in homologous recombination pathway of DNA double‐strand breaks,” PubPeer, December 2025. https://pubpeer.com/publications/98FCE4F5015BC17AF8524FDC9264EC.

