# Supplementary figures and images for: Overexpression of TNKS1BP1 in lung cancers and its involvement in homologous recombination pathway of DNA double‐strand breaks
Source: Cancer Med. 2017 Jan 6;6(2):483–93. doi: 10.1002/cam4.995 (PMC5313643; doi:10.1002/cam4.995)

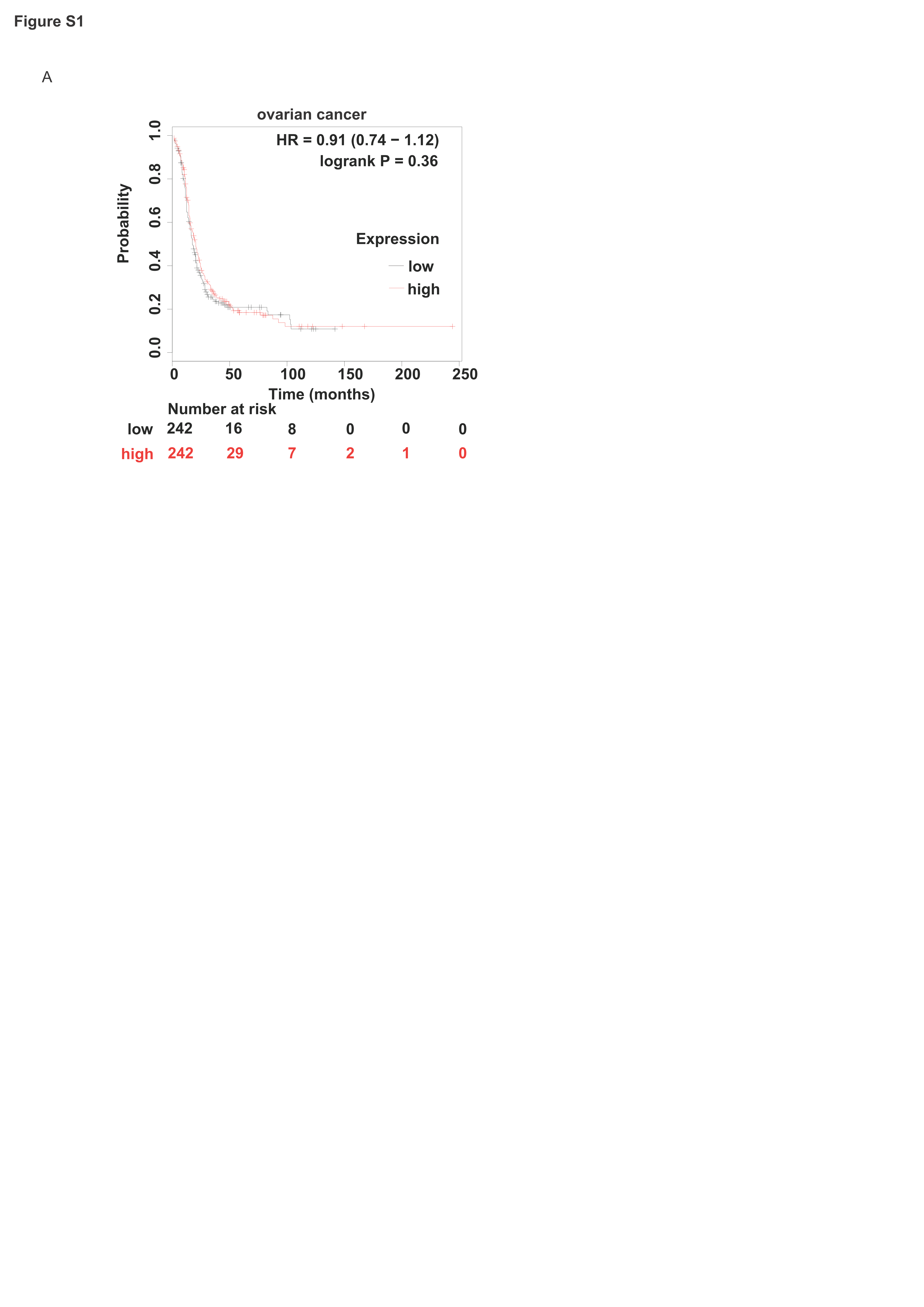

Supplement: Supplementary file 1 — Figure S1. TNKS1BP1 expression was correlated with outcomes in ovarian cancer. [file CAM4-6-483-s001.tiff]

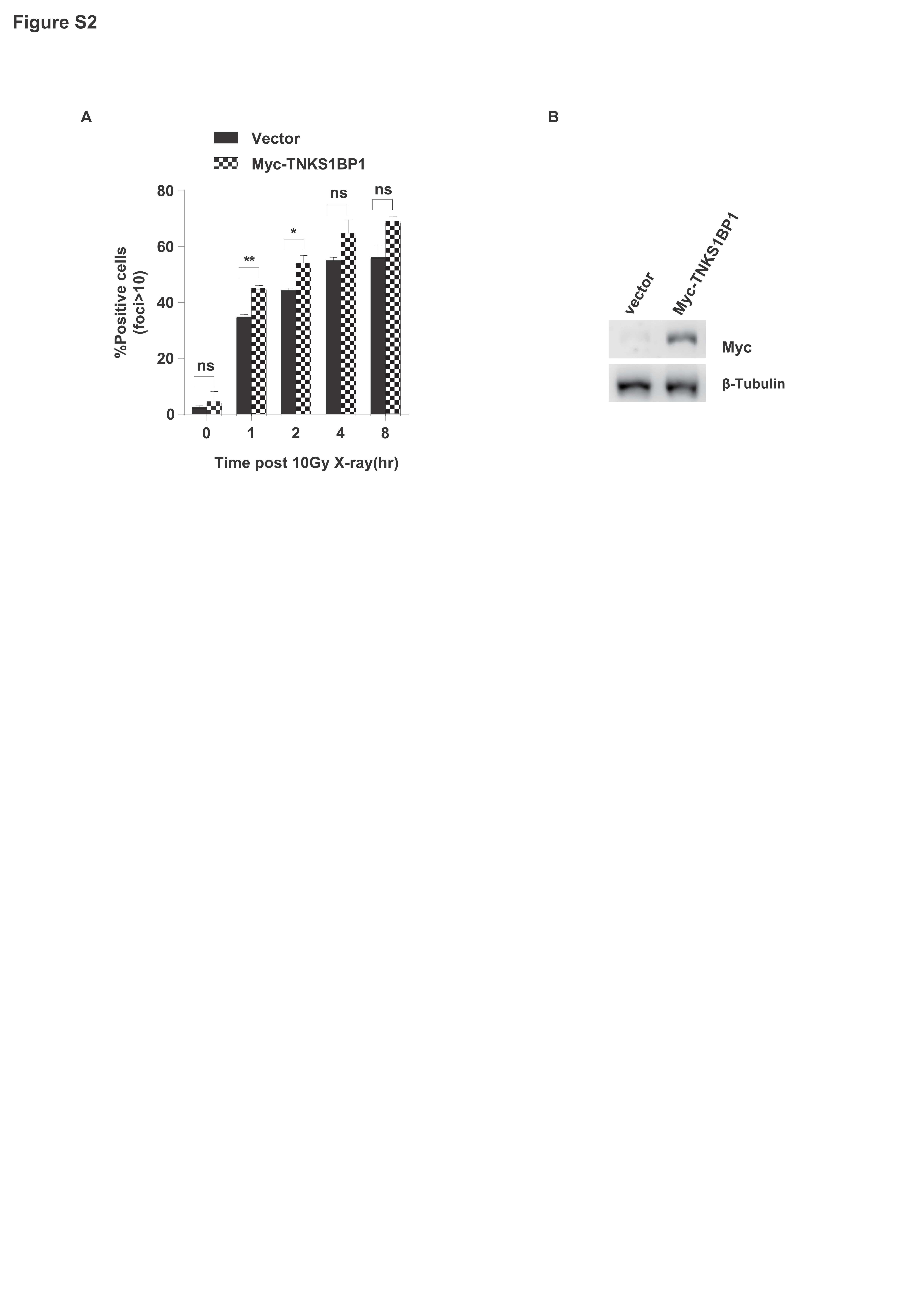

Supplement: Supplementary file 2 — Figure S2. TNKS1BP1 overexpression increases the BRCA1 foci. (A) BRCA1 foci formation following DNA damage was increased in TNKS1BP1 overexpression cells. The cells were treated with ionizing radiation (10 Gy) and allowed to recover for indicating time points. The data were presented as the mean±SEM from two independent experiments. More than 50 cells were counted in each experiment. Data were analyzed by Student's t test (n = 2, two‐sided, *P < 0.05, **P < 0.01). (B) TNKS1BP1 overexpression was confirmed by western blotting analysis in A549 cell. [file CAM4-6-483-s002.tiff]
